# PR and QRS interval changes after transcatheter pulmonary valve replacement in children

**DOI:** 10.1186/s43044-023-00394-x

**Published:** 2023-07-24

**Authors:** Abdelmonem M. Helal, Haysam A. Baho, Ahmed F. Elmahrouk, Mohamed H. Mashali

**Affiliations:** 1 Pediatric Cardiology Department, King Faisal Specialist Hospital and Research Center, Jeddah, Saudi Arabia; 2 Department of Pediatrics, Pediatric Cardiology Division, Cairo University, Cairo, Egypt; 3 Division of Cardiac Surgery, Cardiovascular Department, King Faisal Specialist Hospital and Research Center, MBC J-16, P.O. Box:40047, 21499 Jeddah, Saudi Arabia; 4 Cardiothoracic Surgery Department, Tanta University, Tanta, Egypt

**Keywords:** Transcatheter pulmonary valve replacement, PR interval, QRS interval, Tetralogy of Fallot

## Abstract

**Background:**

Changes in PR intervals after transcatheter pulmonary valve replacement (TCPVR) have not been thoroughly evaluated in children. This study evaluated the changes in PR and QRS intervals six months after TCPVR in children with congenital heart disease.

**Results:**

This study included 41 patients who underwent TCPVR from 2010 to 2022. ECG of patients was reviewed before and six months after TCPVR, and the PR and QRS intervals were reported. Right ventricular systolic pressure (RVSP) was retrieved indirectly from echocardiography and compared pre- and 6-months after TPVR. The median age was 13 years (25th–75th percentiles: 11–16), and 61% were males. The preoperative diagnosis was tetralogy of Fallot (*n* = 29, 71%), transposition of great vessels (*n* = 4, 10%), common arterial trunk (*n* = 3, 7%), pulmonary valve stenosis (*n* = 3, 7%) and pulmonary atresia (*n* = 2, 5%). The Melody valve was used in 30 patients, and Edwards Sapien was used in 11 patients. RVSP was significantly reduced six months after the procedure (pre-RVSP 40 (30–55) mmHg vs. post-RVSP 25 (20–35) mmHg; *P* < 0.001). The PR interval was 142 (132–174) msec before TPVR and 146 (132–168) msec post-TCPVR (*P* = 0.442). Post-TPVR PR was positively related to the pre-PR (*β*: 0.79 (0.66–0.93), *P* < 0.001) and inversely related to the right ventricular outflow tract size (− 1.48 (− 2.76 to − 0.21), *P* = 0.023). The pre-TPVR QRS was 130 (102–146) msec, and the post-TPVR QRS was 136 (106–144) msec (*P* = 0.668).

**Conclusions:**

In children undergoing TCPVR, the PR and QRS intervals did not change significantly during a 6-month follow-up.

## Background

Progressive pulmonary regurgitation (PR) can occur after surgical correction of several congenital cardiac lesions [1, 2]. Pulmonary regurgitation leads to progressive right ventricular (RV) dilatation and dysfunction, consequently leading to a progressive increase in PR degree [3]. Right ventricular dilatation is a major cause of ventricular arrhythmia, right-side heart failure, and sudden cardiac death [4]. Patients with corrected tetralogy of Fallot (TOF) and PR are prone to atrial arrhythmia, decreased quality of life, and survival [5]. Transcatheter pulmonary valve replacement (TCPVR) has emerged to manage pulmonary valve regurgitation after correction of tetralogy of Fallot and other conotruncal anomalies [6, 7]. A wide QRS interval is a risk factor for ventricular arrhythmia and sudden cardiac death in patients with repaired TOF, while its role in predicting atrial arrhythmia is limited [8]. Recent reports indicated that cardiac conduction abnormalities in the form of prolonged PR interval and first-degree heart block were associated with heart failure in patients with right ventricular cardiomyopathy [9, 10]. Studies on the changes in PR intervals after TCPVR in children are limited. Thus, we evaluated the changes in PR and QRS intervals six months after transcatheter pulmonary valve implantation in children with congenital heart disease.

## Methods

### Design

This retrospective cohort study included 41 patients with congenital cardiac lesions who underwent TCPVR from 2010 to 2020 in a single center. We included patients under 18 years of age who had pulmonary regurgitation after a prior correction of congenital heart disease. Patients with preprocedural heart block and those with permanent pacemakers were excluded from the study. Patients with missing baseline or follow-up ECG were also excluded. The study was approved by the local Institutional Review Board, and the need for the patient's (or guardian's) consent was waived.

### Study data

We described the age, weight, height, and body surface area at the time of the procedure. Additionally, we reported the gender, associated genetic disease, diagnosis, and symptoms. Data related to the right ventricular outflow tract conduit, size, degree of pulmonary regurgitation, pulmonary artery pressure, and left ventricular function were recorded. Two types of transcatheter pulmonary valves were used during the study period: Melody (Medtronic Inc.) and Edwards Sapien (Edwards Lifesciences Inc.). The valve's choice depended on the diameter of the right ventricular outflow tract (RVOT). The Edwards Sapien valve was used in patients with large RVOT diameters because of the availability of large valve sizes.

### ECG and outcomes

Twelve-lead ECG was reported before TCPVR and six months after the procedure. PR and QRS intervals were captured from the ECG, and right ventricular systolic pressure (RVSP) was recorded from the baseline and follow-up echocardiography. ECG measurements were reviewed by two cardiologists who were unaware of the patient's data. The measurements of only one physician were recorded, and the measurements from the second were used as confirmatory measures only. A third physician reviewed any controversy between the two measurements. The changes in these parameters were compared between the two measurements.

### Techniques of TCPVR

All patients underwent trans-femoral pulmonary valve replacement. Melody and Sapien valves are balloon-expandable valves. Both valves were delivered using the manufacturer-specific delivery system. The choice of valve type was made according to the discretion of the treating physician.

### Statistical analysis

Descriptive analysis was used to present our results. Normally distributed variables are presented as the mean and standard deviation, and nonnormal variables are presented as the median and interquartile limits. Categorical variables were described as numbers and percentages. The pre- and postprocedure PR and QRS intervals and right ventricular systolic pressure were compared using the Wilcoxon signed rank test for matched pairs. The measures were compared between patients with Melody vs. Sapien valves using the Mann‒Whitney test. Spearman correlation was used to evaluate the relationship between right ventricular pressure and ECG intervals at follow-up. Quantile regression was used to study factors affecting the postprocedural PR interval. All preprocedural variables were included in a stepwise regression model with a forward selection method, and a stay P value was required to retain the variables in the final model. Stata 17 (Stata Corp-College Station-TX-USA) was used for all analyses. A P value of less than 0.05 was considered statistically significant.

## Results

### Preprocedural data

The median age was 13 years (25th–75th percentiles: 11–16), and 61% were males. Two patients had trisomy 21, and one patient had DiGeorge syndrome. The preoperative diagnosis was tetralogy of Fallot (*n* = 29, 71%), transposition of great vessels (*n* = 4, 10%), common arterial trunk (*n* = 3, 7%), pulmonary valve stenosis (*n* = 3, 7%) and pulmonary atresia (*n* = 2, 5%). RVOT was patched in 16 patients (39%), replaced with Contegra (Medtronic's Contegra, Medtronic, Inc.) in 15 patients (37%), a homograft in two patients (5%), or a bioprosthetic valve in four patients (10%) and was native in four patients (10%). Thirty-three patients (80%) had severe pulmonary regurgitation, and 31 (76%) had normal left ventricular function. The Melody valve was used in 30 patients, and Edwards Sapien was used in 11 patients (Table 1).

**Table 1:** Baseline patient characteristics before transcatheter pulmonary valve replacement

Variables	(*n* = 41)
Age (years) [median (Q1–Q3)]	13 (11–16)
Males, n (%)	25 (60.98%)
Weight (kg) [median (Q1–Q3)]	39 (29–54)
Height (cm) [mean ± SD]	147.10 ± 14.61
Body surface area (m2) [mean ± SD]	1.30 ± 0.32
Genetic disease, *n* (%) None Trisomy DiGeorge Others	35 (85.37%) 2 (4.88%) 1 (2.44%) 3 (7.32%)
Diagnosis, n (%)	
Tetralogy of Fallot Common arterial trunk Pulmonary atresia Pulmonary valve stenosis Transposition of great vessels	29 (70.73%) 3 (7.32%) 2 (4.88%) 3 (7.32%) 4 (9.76%)
RVOT type, n (%)	
Native Patched Bioprosthetic Homograft Contegra	4 (9.76%) 16 (39.02%) 4 (9.76%) 2 (4.88%) 15 (36.59%)
Right ventricular outflow tract size (mm) [median (Q1–Q3)]	20 (18–24)
Right ventricular outflow tract lesion, n (%)	
Regurgitation Mixed	17 (41.46%) 24 (58.54%)
NYHA class, n (%)	
I II	33 (80.49%) 8 (19.51%)
Pulmonary regurgitation, n (%)	
Mild Moderate Severe	5 (12.20%) 3 (7.32%) 33 (80.49%)
RV-Pulmonary pressure gradient (mmHg) [median (Q1–Q3)]	40 (10–55)
Left ventricular function, n (%)	
Normal 55–45% < 45%	31 (75.61%) 8 (19.51%) 2 (4.88%)
Type of valves, n (%)	
Melody Edwards Sapien	30 (73.17%) 11 (26.83%)

NYHA: New York Heart Association; RV: right ventricle; RVOT: right ventricular outflow tract

There were no differences in the baseline PR intervals [142 (130–180) vs. 146 (134–156) msec; *P* > 0.99] and QRS interval [125 (102–146) vs. 142 (92–150); *P* = 0.576] between Melody and Sapien valves, respectively. Patients with Sapien valves had significantly lower RVSP than those with Melody valves [45 (30–60) vs. 30 (30–40) mmHg; *P* = 0.021].

### Changes in PR, QRS, and right ventricular pressure

**Fig. 1:**
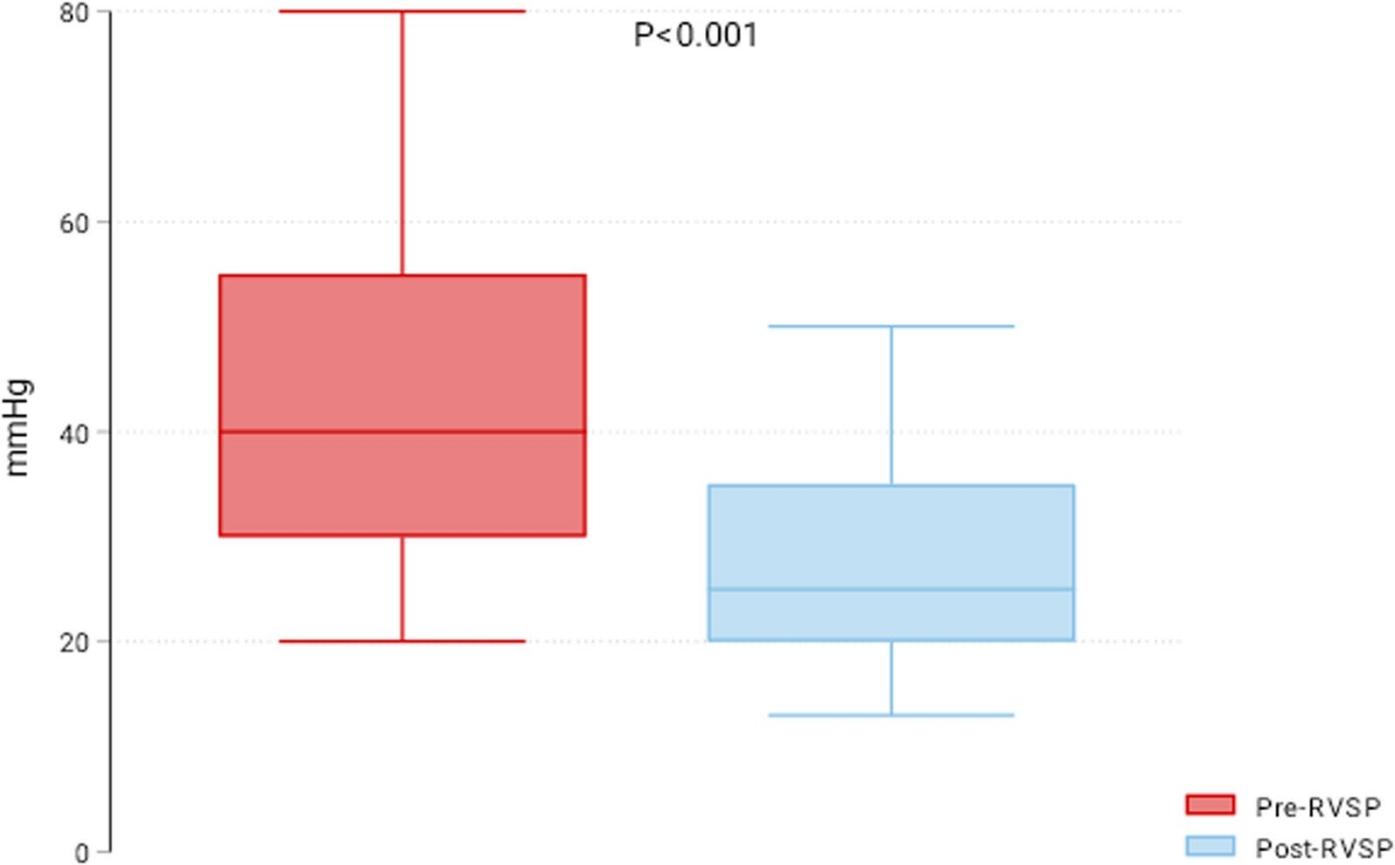
Box plot of right ventricular systolic pressure (RVSP) pre- and six months post-transcatheter pulmonary valve replacement

**Fig. 2:**
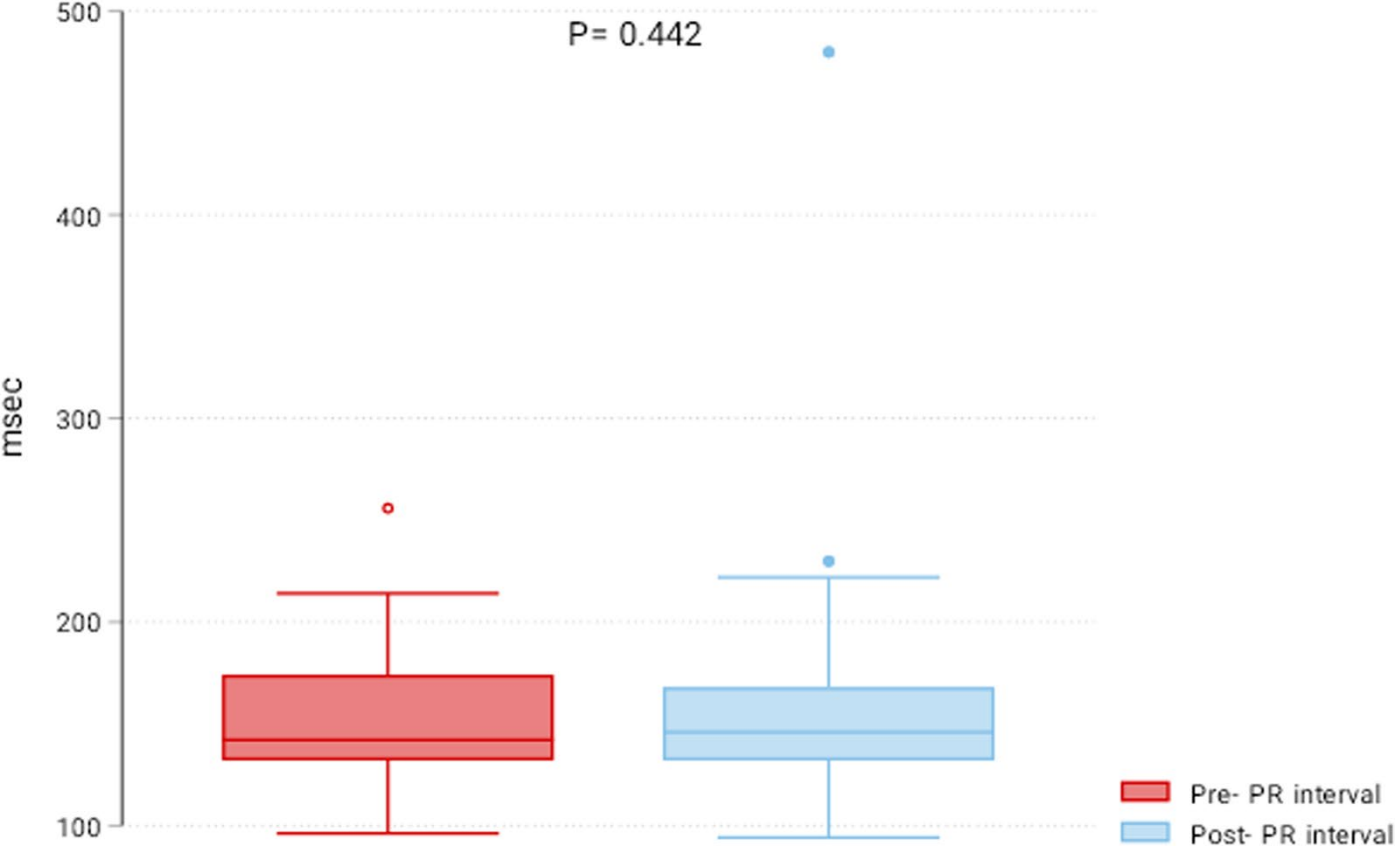
Box plot of PR interval pre- and six months post-transcatheter pulmonary valve replacement

**Fig. 3:**
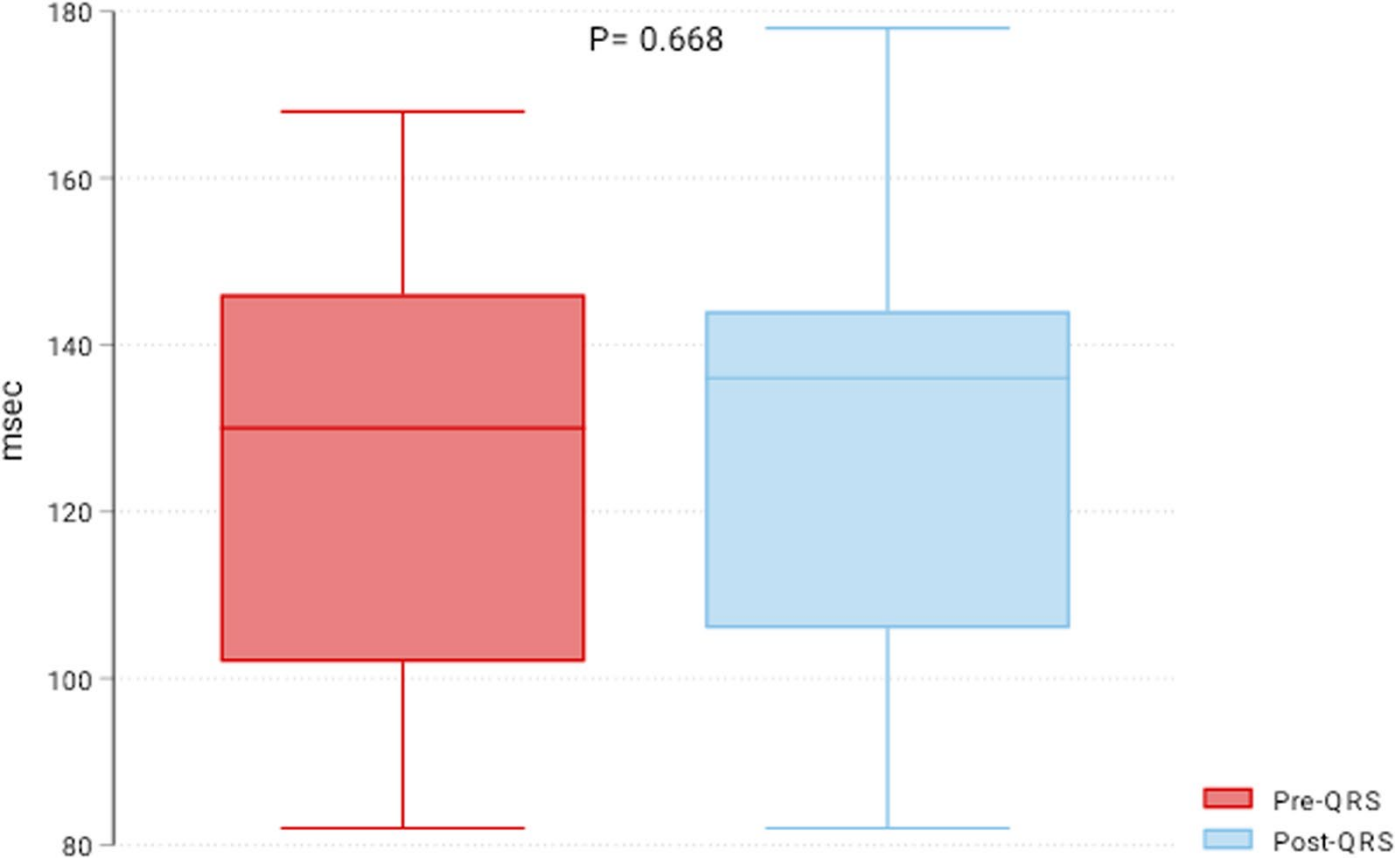
Box plot of QRS interval pre- and six months post-transcatheter pulmonary valve replacement

The RVSP was significantly reduced six months after the procedure (pre-RVSP 40 (30–55) mmHg vs. post-RVSP 25 (20–35) mmHg; *P* < 0.001) (Fig. 1). The PR interval was 142 (132–174) msec before TCPVR and 146 (132–168) msec post-TCPVR (*P* = 0.442) (Fig. 2). The postprocedural PR was positively related to the pre-PR (β: 0.79 (0.66–0.93), *P* < 0.001) and inversely related to RVOT size (− 1.48 (− 2.76 to − 0.21), *P* = 0.023). The preprocedural QRS was 130 (102–146) msec, and the postprocedural QRS was 136 (106–144) msec (*P* = 0.668). The only factor affecting postprocedural QRS was the preprocedural QRS interval. There was no correlation between the PR interval and RVSP (r = − 0.22, *P* = 0.160) or between the QRS interval and RVSP (r = 0.185, *P* = 0.246) at six months after TPVR. There were no differences between both valves regarding the six-month PR interval [146 (132–172) vs. 148 (132–160) msec; *P* = 0.669), QRS interval [133 (106–144) vs. 138 (92–148); *P* = 0.648] and RVSP [30 (20–40) vs. 20 (20–30) mmHg; *P* = 0.06] in Melody vs. Sapien valves, respectively (Fig. 3).

## Discussion

Right ventricular dilatation and dysfunction are commonly associated with pulmonary valve regurgitation [11]. Both can lead to right-side heart failure, conduction abnormalities, arrhythmia, and sudden cardiac death [12]. PR interval prolongation is an indication of abnormal conduction. In recent studies, progressive PR prolongation could be an ominous sign of right ventricular dysfunction and poor outcomes in patients with pulmonary regurgitation [9]. Transcatheter pulmonary valve replacement is increasingly used for managing pulmonary regurgitation in pediatric patients after correcting several congenital cardiac defects [13]. TCPVR has become a feasible and safe option to avoid open heart surgery and interrupt the vicious cycle of pulmonary regurgitation and right ventricular dilatation [14]. Progressive PR prolongation after TCPVR was evaluated in adult patients. This study evaluated the changes in PR and QRS intervals and RVSP six months after TCPVR in children with congenital heart disease. Despite the significant reduction in the right ventricular systolic pressure, we did not report significant changes in PR and QRS intervals.

Several studies evaluated the changes in PR intervals after TCPVR. Kimura and colleagues reported temporal prolongation of PR intervals in adult patients with corrected TOF, which was correlated with right ventricular volumes and function [9]. Massin and coworkers reported a progressive increase in PR and QRS intervals after correcting TOF in 35 patients. The increase in PR interval was more evident in patients with a transannular patch or pulmonary homograft [15]. Sherptong and associates reported a negative correlation between QRS duration before and after pulmonary valve replacement and ventricular arrhythmia, heart failure, and death [16]. Additionally, they found that patients with prolonged QRS intervals before the procedure were less prone to QRS duration reduction after the procedure. Bokma and coworkers reported that QRS fragmentation is superior to QRS duration in predicting mortality after TOF correction [17]. On the other hand, Oosterhof and colleagues showed that the beneficial effect of pulmonary valve replacement on QRS duration was transient, followed by a steady increase in QRS duration only in patients with a preoperative QRS duration of more than 150 ms [18]. In our study, only four patients had QRS durations above 160 ms before TCPVR.

We reported nonsignificant changes in PR and QRS intervals after TCPVR in patients with corrected congenital lesions, mainly TOF. Similarly, Nguyen and coworkers reported no significant difference in QRS duration before and after TCPVR [19]. Additionally, we reported no correlation between ECG intervals and RVSP. These negative results and the difference between our study and other studies could be attributed to several factors. The duration of follow-up may not be sufficient to detect changes in ECG intervals. We followed the patients for six months; however, three months was enough in other studies to detect changes in PR intervals after TOF repair [9]. Furthermore, we included several types of congenital lesions, which could create heterogeneity in our population. However, we performed multivariable analysis for factors affecting the postprocedural PR intervals, and the diagnosis had no association with the postprocedure PR interval. Last, the published studies reported changes in PR intervals in adults with corrected TOF. Our study included pediatric patients only; the changes in PR intervals could require longer follow-up to adulthood. As reported by Oosterhof, the need for a longer follow-up period, even in adulthood, could be essential to show if the reported beneficial effect of TCPVR on QRS duration is transient [18]. The temporal changes in ECG intervals before TPVR are unknown, and TCPVR could have halted the progression of PR or QRS intervals that was reported by kimura and colleagues [9].

## Study limitations

The study has several limitations. The study is a retrospective study with inherent selection bias. The study included patients with TCPVR only, which may not accurately reflect the population with corrected TOF. The small number of patients also limits the study, and we did not correlate the changes in ECG intervals with complications, such as heart failure, ventricular arrhythmia, and mortality.

## Conclusions

In children undergoing TCPVR, the PR and QRS intervals did not change significantly during a 6-month follow-up.

## Data Availability

Un-identified data are available upon request with the corresponding author.

## Abbreviations

PR: Progressive pulmonary regurgitation
RVSP: Right ventricular systolic pressure
TCPVR: Transcatheter pulmonary valve replacement
RV: Right ventricular
TOF: Tetralogy of Fallot
RVOT: Right ventricular outflow tract
